# A high‐dose, depigmented polymerized birch pollen extract for subcutaneous allergen immunotherapy has a favourable efficacy/safety ratio

**DOI:** 10.1002/clt2.12315

**Published:** 2023-11-15

**Authors:** Oliver Pfaar, Angelika Sager, Ralph Mösges, Margitta Worm

**Affiliations:** ^1^ Department of Otorhinolaryngology, Head and Neck Surgery, Section of Rhinology and Allergy University Hospital Marburg Philipps‐Universität Marburg Marburg Germany; ^2^ LETI Pharma GmbH Witten Germany; ^3^ ClinCompetence Cologne GmbH Cologne Germany; ^4^ Institute of Medical Statistics and Computational Biology Faculty of Medicine University of Cologne Cologne North Rhine‐Westphalia Germany; ^5^ Division of Allergy and Immunology Department of Dermatology, Venereology and Allergy Charité‐Universitätsmedizin Berlin Berlin Germany

**Keywords:** allergen immunotherapy, allergic rhinoconjunctivitis, birch pollen allergy, conjunctival provocation test

## Abstract

**Background:**

Subcutaneous allergen immunotherapy (SCIT) with depigmented, polymerized (DPP) birch pollen extract has been marketed at doses of up to 1000 DPP units/mL since 2001. We sought to determine the dose‐dependent efficacy of a DPP birch pollen extract formulation in patients suffering from birch‐pollen‐induced allergic rhinitis or rhinoconjunctivitis with or without intermittent asthma.

**Methods:**

A titrated conjunctival provocation test (CPT) was applied as a surrogate marker. This Phase II randomized, double‐blind, parallel‐group, dose‐ranging clinical trial was performed at 39 centres in Germany, Lithuania and Poland. After randomization to four dose‐level groups (100, 1000, 5000 and 10,000 DPP units/mL) and up‐dosing, participants received maintenance SCIT with five monthly subcutaneous injections. The primary endpoint was the proportion of patients in whom a higher concentration of birch pollen (vs. baseline) was needed to elicit a positive CPT.

**Results:**

Three hundred forty‐three patients were included (mean (range) age: 42.6 (19–70)). The highest CPT responder rates were seen in the higher dose‐level groups. In the intention‐to‐treat analysis, the difference between the 100 and 10,000 groups was statistically significant (*p* = 0.0118). Although the proportion of patients with ≥1 treatment‐emergent adverse events increased with the dose, almost all these events were mild (65.6%) or moderate (18.5%).

**Conclusion:**

Judging by the results of a CPT, the efficacy/safety ratio in SCIT appears to be favourable for a high‐dose‐level preparation of a DPP birch pollen extract.

## 1 BACKGROUND

Allergic rhinoconjunctivitis and allergic asthma induced by outdoor and/or indoor aeroallergens are increasingly prevalent worldwide.^1^, ^2^ Along with underlying etiological trends in the occurrence of allergic disease, climate change and exposure to urban air pollutants are accentuating the pathological effects of pollen allergens.^3^ Birch (*Betula*) pollen allergy is a notable health problem in northern and central Europe, as the duration and intensity of the birch pollen season increases.^4^ Symptomatic medications may lack efficacy or be poorly tolerated in some cases of moderate‐to‐severe allergic rhinoconjunctivitis; allergen immunotherapy (AIT, whether administered as subcutaneous allergen immunotherapy (SCIT) or sublingual allergen immunotherapy (SLIT)) is the only disease‐modifying treatment for allergic respiratory diseases.^5^, ^6^, ^7^, ^8^, ^9^ A variety of birch pollen SCIT or SLIT formulations have been shown to be efficacious and safe in patients with moderate‐to‐severe birch pollen allergy.^10^, ^11^, ^12^ The risk‐benefit ratio of an allergen extract depends notably on its allergenicity (i.e. the induction of an abnormal allergic reaction, with potential functional impairments and/or tissue damage) and its immunogenicity (i.e. the ability to stimulate and modify immune responses and systems). It has been shown that physically or chemically modified allergen extracts (sometimes referred to as “allergoids”) lose their allergenicity but retain their immunogenicity (relative to native extracts) and are therefore suitable for use in AIT.^13^, ^14^, ^15^ In particular, depigmented polymerized (DPP) allergen extracts can be prepared by treatment with mild acid (to remove low molecular weight compounds: depigmentation) and protein crosslinking with glutaraldehyde (polymerization); these extracts have been extensively characterized and approved for safe, effective SCIT and/or SCIT in allergic respiratory diseases induced by birch pollen, grass pollen, and house dust mite aeroallergens.^10^, ^16^, ^17^, ^18^, ^19^, ^20^, ^21^, ^22^, ^23^, ^24^, ^25^

Prior to the study, the DPP birch pollen extract for SCIT investigated here had been marketed in Germany at doses of up to and including 1000 DPP/mL (corresponding to ∼60 μg Bet v 1/mL prior to depigmentation).^10^, ^22^, ^23^, ^24^ The Phase II study described here was part of the clinical development of a DPP birch pollen formulation under the German Therapy Allergen Ordinance.^26^, ^27^

Hence, the primary objective of the present study was to determine the optimal dose of a DPP birch pollen extract formulation (up to 10,000 DPP/mL) in adults with birch‐pollen‐induced allergic rhinitis or rhinoconjunctivitis with or without intermittent asthma, as judged by the results of a conjunctival provocation test (CPT).^28^, ^29^, ^30^ The secondary objectives were to determine immunological changes and to assess safety (as judged by the occurrence of local reactions (LRs) and systemic reactions (SRs)).

## 2 METHODS

### 2.1 Trial design

We performed a Phase II multinational, multicentre, randomized clinical trial with four parallel groups in 39 study centres located in Germany, Lithuania, and Poland. The study ran from May 10th, 2010 (first visit (V) by the first patient) to February 21st, 2011 (last visit by the last patient), that is, after the 2010 birch pollen season and before the 2011 season in the three countries. After a 2‐week screening phase (V1), eligible patients were randomized 1:1:1:1 to the four dose levels of the DPP birch pollen extract (see below). The study then comprised a 1‐day rush up‐dosing phase (V2), a maintenance phase with five roughly monthly subcutaneous injections (in the upper arm) of the allotted DPP birch pollen extract formulation during V3 to V7 (see below for more details) and an end‐of‐study V8. The total study duration per patient was about 6 months.

### 2.2 Trial population

The study's main inclusion criteria were as follows: (i) age from 18 to 70 at the time of the first (screening) study visit; (ii) self‐reported perceived disease activity of at least 30 mm on a 0–100 mm visual analogue scale; (iii) a forced expiration volume (FEV1) or highest of three peak exploratory flow values > 80% of the predicted normal value, (iv) a self‐reported history of allergic rhinitis and/or rhinoconjunctivitis symptoms for at least the previous 2 years (with or without intermittent asthma symptoms) and caused by physician‐diagnosed sensitization against birch pollen; (v) IgE‐mediated sensitization to birch pollen, as documented by a suggestive medical history, the presence of specific IgE (sIgE) against birch pollen (CAP ≥2 kU/L), a positive skin prick test (SPT: positive if the wheal diameter was ≥3 mm and at least the size of a histamine reference test) to birch pollen extract, and (vi) a positive CPT (see below) with a birch pollen concentration of up to 10,000 standard quality units (SQ‐U)/mL.

The main exclusion criteria were as follows: (i) acute or chronic conjunctivitis, (ii) a history of significant clinical manifestations of allergy as a result of sensitization against grass or weed pollen and perennial allergens (e.g. house dust mites), (iii) persistent asthma (as defined by the 2007 version of the Global Initiative for Asthma (GINA) guidelines),^31^ (iv) birch pollen AIT within the previous 5 years, and (vi) the standard contraindications to AIT.^8^, ^32^, ^33^

### 2.3 The conjunctival provocation test

The CPT is a validated, highly reproducible method for measuring an individual's sensitivity to an allergen; in the present study, the CPT was performed according to Möller's procedure.^28^, ^29^ The tested birch pollen concentrations were 100, 330, 1,000, 3300 and 10,000 SQ‐U/mL (in that order) at V1, and 100, 330, 1,000, 3,300, 10,000, 33,000, and 100,000 SQ‐U/mL at V8. A higher concentration was applied only if the result of the immediately preceding test was negative (see below). Initially, a single drop of 100 SQ‐U/mL was placed in the left conjunctival sac. The right conjunctival sac was used as the reference. Ten minutes after applying the drop, the intensity of four individual symptoms (eye redness; weeping; itching or burning; runny or blocked nose) were rated (0 = absent, 1 = mild, 2 = moderate, 3 = severe). The individual symptom scores were summed to give a total symptom score (i.e. from 0 to 12). A positive CPT was defined as a test that resulted in a total symptom score of five or more for any of the allergen concentrations administered to the eye. The procedure was stopped once a positive CPT had been observed. All study sites were provided with a detailed instruction manual on the performance of the CPT.

### 2.4 The study treatment and randomization

The birch pollen preparation for SCIT was Depigoid® Birch (Laboratorios LETI, Barcelona, Spain) adsorbed onto aluminium hydroxide and suspended in 0.9% NaCl with 0.5% phenol. The 5000 DPP/mL dose corresponded to a titre of ∼300 μg Bet v1/mL prior to depigmentation. For each of the four dose levels (100, 1,000, 5000 and 10,000 DPP/mL), the treatment regimen comprised a rush (1‐day) build‐up phase with three injections (0.1 mL, 0.2 and 0.2 mL of the allotted formulation) and a 22‐week maintenance phase with five 0.5 mL injections at 3–4‐week intervals.

After each injection, the patient was observed by the study personnel for at least 30 min. If the patient had a lung function text result ≤80% of the normal value or developed a severe LR (as judged by the investigator with regard to the induration size, itching, and pain) or an SR of any kind (according to the 1993 guidelines issued by the European Academy of Allergology and Clinical Immunotherapy),^34^ the reaction was treated and the patient was excluded from the study.

Each medication package was identified by a unique, computer‐generated, three‐digit medication number printed on the label, corresponding to the randomization number. The randomization schedule was generated by dedicated software and was not known to the investigators or other study personnel. Study participants were provided with (and instructed in the use of) antihistamine tablets, eyedrops and nasal spray, prednisolone tablets, and a salbutamol inhaler.

### 2.5 Endpoints and assessments

The primary efficacy endpoint was the proportion of patients in each dose‐level group with an increase in the concentration of allergen needed to elicit a positive CPT at the end of the treatment period, relative to measurements at baseline (referred to hereafter as the CPT responder rate).

The secondary efficacy endpoints were immunological laboratory variables (total serum IgE, specific IgE (s‐IgE), s‐IgG_1_, and s‐IgG_4_) and individual patient CPT results (i.e. V8 vs. V1 CPT results). The safety endpoints were standard haematology and clinical chemistry variables, adverse events (AEs), a patient diary and an overall evaluation (by the patient and by the physician) of the safety of the AIT (excellent, good, moderate, poor, or unacceptable).

Adverse events were coded according to version 13.1 of the Medical Dictionary for Regulatory Activities (MedDRA: https://www.meddra.org/).

### 2.6 Sample size calculation and statistical analysis

The sample size calculation was based on the assumption that 60% of the patients in the 1000 DPP/mL group would have a change in the CPT test result (i.e. an increase in the lowest triggering allergen concentration). With a one‐sided alpha risk of 2.5% in Fisher's exact test, a sample size of 70 patients per group would give a power of 80%. Assuming a dropout rate of at least 10%, the inclusion of a total of 320 patients (i.e. 80 per group) was required. Continuous variables were expressed as the mean (range) or mean (standard deviation (SD)) when normally distributed or the median [interquartile range (IQR)] when not. Categorical variables were expressed as the frequency (percentage). All statistical analyses were performed using SAS® software (version 9.2, SAS Institute Inc.). For intergroup comparison of the responder rate, the threshold for statistical significance was set at *p* < 0.025.

### 2.7 Ethics

All participants gave their written consent for participation after being informed of the study objectives and procedures. The study was approved by the appropriate independent ethics committees in each country and registered in the European Clinical Trials Database (reference: EudraCT 2008‐008448‐26). The study was carried out in accordance with the Declaration of Helsinki and the International Conference on Harmonisation E6 guideline on good clinical practice (CPMP/ICH135/95) and the appropriate local regulations and legislation.

## 3 RESULTS

### 3.1 Study participants

**FIGURE 1:**
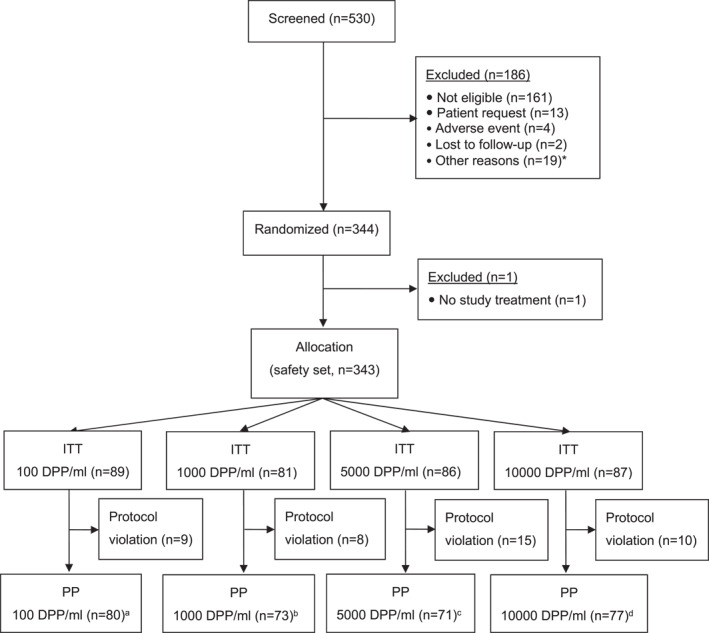
The patient disposition. ITT, intention‐to‐treat; PP, per‐protocol. *more than 1 reason per patient possible, ^a^including 3 patients classified as dropouts, ^b^including 4 patients classified as dropouts, ^c^including 2 patients classified as dropouts, ^d^including 3 patients classified as dropouts.

A total of 530 patients were screened, and 344 were randomized at 39 study sites (Figure 1).

One randomized patient did not receive any study treatment. The safety and the intention‐to‐treat (ITT) populations both comprised 343 patients (148 men (43.1%) and 195 women (56.9%); 100 DPP/mL: *n* = 89; 1000 DPP/mL: *n* = 81; 5000 DPP/mL: *n* = 86; 10,000 DPP/mL: *n* = 87). Forty‐two patients had major protocol deviations, and so the per‐protocol (PP) population comprised 301 patients. Twenty‐nine patients withdrew prematurely from the study, mainly due to AEs.

When considering the safety population, there were no significant differences in demographic and clinical characteristics between the dose‐level groups (Table 1).

**TABLE 1:** Demographic, clinical and immunological characteristics of the patients in the four dose‐level groups.

Variable	Dose level group			
	100 DPP/ml (*n* = 89)	1000 DPP/mL (*n* = 81)	5000 DPP/mL (*n* = 86)	10,000 DPP/mL (*n* = 87)
Women	49	41	55	50
Men	40	40	31	37
Age (years)	43.24 ± 12.270	42.15 ± 12.610	44.03 ± 13.215	41.05 ± 11.155
Height (cm)	171.6 ± 9.55	172.5 ± 9.52	169.8 ± 9.19	171.2 ± 9.39
Weight (kg)	74.5 ± 15.21	77.0 ± 15.00	75.0 ± 16.65	76.4 ± 15.30
Regular smokers	6 (6.7%)	6 (7.4%)	3 (3.5%)	8 (9.2%)
Hypertension	10 (11.2%)	8 (9.9%)	14 (16.3%)	10 (11.5%)
Asthma (always intermittent)	10 (11.2%)	8 (9.9%)	8 (9.3%)	13 (14.9%)
Concomitant medications	63 (70.8%)	53 (65.4%)	72 (83.7%)	60 (69.0%)
Birch ss‐IgE at V1 (kU/L)	65.5 ± 33.56	65.1 ± 35.31	64.2 ± 36.52	67.3 ± 33.98)
Total serum IgE at V1 (mg/L)	0.50 ± 1.652	0.47 ± 1.037	0.44 ± 1.077	0.46 ± 0.812
Lowest allergen concentration for a positive CPT at V1 (SQ‐U/mL)	4091 ± 3773.6	4163 ± 3857.6	4010 ± 3911.8	4580 ± 4089.3

*Note*: The data are quoted as the mean ± SD or *n* (%).

Abbreviations: CI, confidence interval; CPT, conjunctival provocation test; DPP, depigmented, polymerized birch pollen extract; birch ss‐IgE, serum specific IgE for birch pollen extract.

Overall, the mean (range) age was 42.6 (19–70) years, and the mean (range) body mass index was 25.7 (17.3–47.8) kg/m^2^. All the patients reported only occasional or no alcohol consumption. The most frequent comorbidity was arterial hypertension in 42 patients (12.2%). All but one of the 343 patients had a positive SPT for birch pollen, and the proportion of patients with a positive SPT was below 30% for the other allergens tested (Supplementary Table S1). The ss‐IgE titres were 20‐ to 130‐fold greater for birch pollen than for the other allergens tested (Supplementary Table S2).

### 3.2 Primary endpoint

Overall, the highest responder rates were seen in the higher dose‐level groups, relative to the comparator (100 DPP/mL) (Table 2). In the ITT population, only the difference between the 100 and 10,000 DPP/mL groups was statistically significant at *p* < 0.025 (*p* = 0.0118). In the PP population, the difference between the 100 and 5000 DPP/mL groups and the difference between the 100 and 10,000 DPP/mL groups were both statistically significant at *p* < 0.025.

**TABLE 2:** Conjunctival provocation test responder rates in the four dose‐level groups (ITT and PP populations).

	Responder rate [95% CI]^a^
ITT population (*n* = 343)	
100 DPP/mL	36.0% [26.1%, 46.8%]
1000 DPP/mL	46.9% [35.7%, 58.3%]
5000 DPP/mL	51.2% [40.1%, 62.1%]
10,000 DPP/mL	54.0% [43.0%, 64.8%]

	Intergroup difference [97.5% CI]	*p*‐value in Fisher's exact test^b^
100 versus 10,000 DPP/mL	−18.1% [−∞, −3.6%]	0.0118
100 versus 5000 DPP/mL	−15.2% [−∞, −0.7%]	0.0301
100 versus 1000 DPP/mL	−11.0% [−∞, 3.8%]	0.0978

	Responder rate [95% CI]^a^
PP population (*n* = 301)	
100 DPP/mL	37.5% [26.9%, 49.0%]
1000 DPP/mL	50.7% [38.7%, 62.6%]
5000 DPP/mL	54.9% [42.7%, 66.8%]
10,000 DPP/mL	55.8% [44.1%, 67.2%]

	Intergroup difference [97.5% CI]	*p*‐value in Fisher's exact test^b^
100 versus 10,000 DPP/mL	−18.3% [−∞, −3.0%]	0.0159
100 versus 5000 DPP/mL	−17.4% [−∞, −1.7%]	0.0236
100 versus 1000 DPP/mL	−13.2% [−∞, 2.4%]	0.0695

Abbreviations: DPP, depigmented, polymerized, birch pollen extract; ITT, intention‐to‐treat; PP, per‐protocol.

^a^ CIs for the responder rates are two‐sided.

^b^ CIs for the difference in responder rates and for Fisher's exact test are one‐sided.

### 3.3 Secondary endpoints

The titre of birch s‐IgE decreased between V1 and V8 in all dose‐level groups, with greater decreases in the lower dose‐level groups (Table 3). In the ITT analysis, the decrease in s‐IgE was significantly greater in the 100 DPP/mL group than in the three other groups. In the PP analysis, the decrease in s‐IgE was significantly greater in the 100 DPP/mL group than in the 5000 and 10,000 DPP/mL groups.

**TABLE 3:** Immunological endpoints: birch‐specific serum IgE and total serum IgE at V1 and V8, with V8‐V1 differences (ITT set, *N* = 343; PP set, *N* = 301).

	100 DPP/mL (*n* = 89)	1000 DPP/mL (*n* = 81)	5000 DPP/mL (*n* = 86)	10,000 DPP/mL (*n* = 87)
ITT population (*n* = 343)				
Birch sIgE (kU/L)				
V1	65.48 ± 33.562	65.05 ± 35.312	64.16 ± 36.520	67.33 ± 33.979
V8	55.46 ± 34.029	58.85 ± 35.104	60.73 ± 36.423	66.44 ± 31.579
V8‐V1	−10.51 ± 14.722	−7.07 ± 13.096	−3.78 ± 12.513	−1.10 ± 11.857
Total serum IgE (mg/L)				
V1	0.50 ± 1.652	0.47 ± 1.037	0.44 ± 1.077	0.46 ± 0.812
V8	0.29 ± 0.495	0.39 ± 1.131	0.39 ± 0.835	0.37 ± 0.656
V8‐V1	−0.05 ± 0.374	−0.07 ± 0.142	−0.05 ± 0.381	−0.07 ± 0.345
PP population (*n* = 301)				
Birch s‐IgE (kU/L)				
V1	66.80 ± 33.545	65.61 ± 34.813	64.68 ± 37.076	69.39 ± 32.851
V8	56.79 ± 34.146	58.11 ± 35.011	59.60 ± 36.594	67.52 ± 32.030
V8‐V1	−10.42 ± 14.977	−7.31 ± 13.393	−4.77 ± 12.764	−1.05 ± 10.916
Total serum IgE (mg/L)				
V1	0.35 ± 0.514	0.48 ± 1.084	0.34 ± 0.433	0.46 ± 0.842
V8	0.30 ± 0.513	0.39 ± 1.162	0.29 ± 0.377	0.39 ± 0.669
V8‐V1	−0.06 ± 0.390	−0.08 ± 0.145	−0.02 ± 0.146	−0.08 ± 0.354

*Note*: The data are quoted as the mean ± SD.

Abbreviations: DPP, depigmented, polymerized, birch pollen extract; ITT, intention‐to‐treat; PP, per‐protocol.

The titres of birch‐specific IgG_1_ and IgG_4_ increased between V1 and V8, with greater increases in the higher DPP dose‐level groups (Supplementary Table S3). The increase was significantly smaller in the 100 DPP/mL group than in the three other groups. The mean overall IgE titres slightly decreased in all groups.

### 3.4 Safety

A total of 834 treatment‐emergent adverse events (TEAEs) were reported in the safety set. Between the first application of the study treatment and the end of the study, 219 (63.8%) of the 343 patients in the safety set experienced one TEAE or more (Table 4). The proportion of patients with one TEAE or more increased with the dose; the differences between the 100 DPP/mL group on one hand and the 5000 and 10,000 DPP/mL groups on the other were statistically significant (*p* = 0.0186 and *p* = 0.0079, respectively), as was the difference between the 1000 and 10,000 DPP/mL groups (*p* = 0.0160). The difference between the 1000 and 5000 groups was not significant (*p* = 0.0356). With regard to intensity, almost all the TEAEs were mild (65.6%), moderate (18.5%) or (for systemic AEs) classified as Grade 1 (124 [14.9%]). There were no severe TEAEs in patients from the 100 and 1000 DPP/mL groups, and five severe TEAEs were observed in patients from the 5000 and 10,000 DPP/mL groups. It noteworthy that asthma was reported as a TEAE in few patients (*n* = 8 in total: 4 in the 100 DPP/mL group, and 2 each in the 5000 and 10,000 DPP/mL groups).

**TABLE 4:** Overview of TEAE (safety set, *n* = 343).

TEAEs		100 DPP/mL (*n* = 89)	1000 DPP/mL (*n* = 81)	5000 DPP/mL (*n* = 86)	10,000 DPP/mL (*n* = 87)
Number of patients with TEAEs		48 (53.9%)	45 (55.6%)	62 (72.1%)	64 (73.6%)
Number of TEAEs		142	116	218	358
Causality assessment by the investigator	Definitely unrelated	53 (37.3%)	36 (31.0)	40 (18.3)	61 (17.0)
	Probably unrelated	22 (15.5)	11 (9.5)	19 (8.7)	27 (7.5)
	Probably related	33 (23.2)	18 (15.5)	46 (21.1)	80 (22.3)
	Definitely related	34 (23.9)	51 (44.0)	113 (51.8)	190 (53.1)
Intensity	Local—Mild	84 (59.2)	72 (62.1)	145 (66.5)	246 (68.7)
	Local—Moderate	19 (13.4)	28 (24.1)	35 (16.1)	72 (20.1)
	Local—Severe	‐	‐	4 (1.8)	1 (0.3)
	Systemic—Grade 1	38 (26.8)	15 (12.9)	34 (15.6)	37 (10.3)
	Systemic—Grade 2	‐	‐	‐	2 (0.6)
	Systemic—Grade 3	‐	‐	‐	‐
	Systemic—Grade 4	‐	‐	‐	‐
	Missing	1 (0.7)	1 (0.9)	‐	‐

Abbreviations: DPP, depigmented, polymerized birch pollen extract; TEAE, treatment‐emergent adverse event.

Considering the safety set, 565 of the 834 TEAEs were considered to be adverse drug reactions, with 388 (46.5%) definitely related to the study treatment and 177 (21.2%) probably related. Overall, the most frequently reported SRs (classified by MedDRA preferred term (PT)) were ‘rhinitis, allergic’ (in 21 patients (6.1%)) and ‘sneezing’ (also in 21 patients (6.1%)). The most frequently reported LR (by MedDRA PT) in all groups was injection site oedema or swelling (Table 5).

**TABLE 5:** Frequently reported TEAEs (numbers of patients in the safety set, *n* = 343), by MedDRA preferred term.

TEAE symptom (MedDRA PT)	100 DPP/mL (*n* = 89)	1000 DPP/mL (*n* = 81)	5000 DPP/mL (*n* = 86)	10,000 DPP/mL (*n* = 87)
Any TEAE symptom	48 (53.9)	45 (55.6)	62 (72.1)	64 (73.6)
Injection site swelling	3 (3.4)	12 (14.8)	22 (25.6)	25 (28.7)
Injection site reaction	3 (3.4)	6 (7.4)	10 (11.6)	19 (21.8)
Injection site pain	4 (4.5)	7 (8.6)	11 (12.8)	20 (23.0)
Injection site oedema	3 (3.4)	4 (4.9)	10 (11.6)	14 (16.1)
Nasopharyngitis	17 (19.1)	5 (6.2)	13 (15.1)	10 (11.5)
Sneezing	9 (10.1)	5 (6.2)	8 (9.3)	10 (11.5)
Rhinitis allergic	6 (6.7)	3 (3.7)	8 (9.3)	8 (9.2)
Rhinorrhoea	6 (6.7)	2 (2.5)	4 (4.7)	5 (5.7)
Injection site pruritus	2 (2.2)	2 (2.5)	7 (8.1)	6 (6.9)
Cough	5 (5.6)	4 (4.9)	5 (5.8)	2 (2.3)
Injection site erythema	1 (1.1)	1 (1.2)	3 (3.5)	5 (5.7)
Vertigo	2 (2.2)	3 (3.7)	4 (4.7)	2 (2.3)
Asthma	4 (4.5)	‐	2 (2.3)	2 (2.3)
Dizziness	1 (1.1)	4 (4.9)	1 (1.2)	4 (4.6)

Abbreviations: DPP, depigmented, polymerized birch pollen extract; MedDRA, Medical Dictionary for Regulatory Activities; PT, preferred term; TEAE, treatment‐emergent adverse event.

No patients died during the course of the study. None of the few serious TEAEs (*n* = 7) were assessed as definitely or probably related to the study treatment. The serious TEAEs that were considered to be unrelated to the study treatment were loss of consciousness, road traffic accident, ventricular extrasystoles, Hodgkin's disease, haemorrhoid operation, breast cancer, acute abdomen, and wrist fracture.

Only a few patients took rescue medication, and no striking differences were observed between the dose‐level groups. The majority of the patients (in all groups) and the investigators rated the overall safety of the study treatment as ‘excellent’ or ‘good’. Only two patients rated the overall safety as ‘poor’ (one in the 100 DPP/mL group and one in the 1000 DPP/mL group). None of the patients in the 5000 and 10,000 DPP/mL groups rated the overall safety as ‘poor’ or ‘unacceptable’.

## 4 DISCUSSION

In the last few decades, AIT has moved into the evidence‐based era of personalized medicine. As such, many AIT formulations are now being tested in robust clinical trials that meet strict regulatory requirements.^35^, ^36^, ^37^ It is now clear that the efficacy and safety of AIT are both governed by dose–response relationships, and therefore, treatment with a proven clinically effective dose is essential. In the conventional clinical development of pharmaceuticals, Phase II corresponds to dose‐ranging; several doses are tested, and the one with the optimal risk‐benefit ratio is selected for further testing in the so‐called ‘pivotal’ Phase III clinical trials considered by the regulatory authorities for marketing authorization. Nevertheless, the field of AIT has been slow to adopt dose‐ranging trials, and most of the dose‐ranging trials have been performed in the last dozen or so years.^24^, ^38^, ^39^, ^40^, ^41^, ^42^, ^43^

The present dose‐ranging study was performed in 2010–2011. A similar Phase II multicentre, randomized, double‐blind, placebo‐controlled, parallel‐group study in Austria, Germany, and Poland was performed in 2017/2018 for a grass pollen allergoid formulation.^44^ The course of SCIT comprised six injections of four dose levels of an allergoid grass pollen extract over 31–41 days. The study results revealed a strong, curvilinear dose‐response curve for the total symptom score in a CPT, which was consistent with the secondary endpoints.

In the present study, we also determined that higher dose levels were associated with a higher CPT responder rate ‐ a finding that was mirrored by the results for the secondary endpoints. Furthermore, the higher dose levels were not associated with a higher frequency of serious TEAEs.

The present study had a number of strengths. Firstly, we used a standardized measurement technique—the CPT—to gauge the dose‐dependent efficacy of the SCIT formulations.^29^, ^30^ It is noteworthy that the CPT's efficacy results were consistent with the patient's subjective evaluations in their diary. Furthermore, it has been reported that early nonreactivity in a CPT reportedly predicts a positive outcome in AIT.^44^, ^45^ Secondly, the study took place outside the birch pollen season, which doubtless reduced interference with the results of the CPT and with safety measurements of reactions to the product's administration. Lastly, the three‐country, 39‐centre study design suggests that the present results can be extrapolated with confidence in populations in Western European countries in general.

The present study also had some limitations. Firstly, although the CPT was standardized, the interpretation of the eye signs and symptoms was probably still somewhat operator‐dependent. To mitigate this source of bias, all investigating centres were provided with a detailed instruction manual. Secondly, the CPT protocol was not validated before its use in the present study. However, we have since validated a CPT protocol based on the methodology of Möller et al.^26^ and others.^46^ In allergology more generally, CPTs are less commonly encountered than nasal provocation tests (NPTs).^47^, ^48^ NPTs can be standardized to a good extent, but the results (as a subjective nasal symptom score and/or an objective nasal air flow rate) are sometimes difficult to interpret.^49^, ^50^ Environment exposure chambers (also referred to as allergen challenge chambers) provide the most ‘natural’ exposure to aeroallergens and are increasingly being used in Phase II to IV trials of allergy therapies but, for logistic reasons, are not as well suited to studies in which the investigating centres are far from the chamber facility.^47^, ^51^, ^52^ A comparison of the CPT with another challenge method would have been interesting but was not logistically possible in the present study. Thirdly, the fact that the study took place outside the birch pollen season could also be considered as a limitation; the effect of SCIT might differ when participants are ‘primed’ or concomitantly exposed to the natural triggering allergen. However, the results of a CPT (or another type of provocation test) performed during the season might also be biased by natural exposure. Furthermore, SCIT for a seasonal allergy usually involves a pre‐seasonal regimen. Fourth, the study lacked a placebo comparator group. However, placebo SCIT can be challenged from an ethical point of view and may not be appropriate in Phase I and II trials. Lastly, we did not look at whether treatment with the DPP birch pollen extract was more effective in monosensitized patients than in polysensitized patients (or vice versa) or had cross‐efficacy on other allergies or sensitization in weakly polyallergic/polysensitized patients. In fact, a history of significant clinical manifestations of allergy as a result of sensitization against grass or weed pollen and perennial allergens was a study exclusion criterion.

In conclusion, the efficacy/safety ratio in SCIT appears to be favourable for a high‐dose‐level preparation of a DPP birch pollen extract: efficacy was significantly greater but the safety profile—even for the highest dose—was acceptable and consistent with the literature data on SCIT with formulations of birch pollen extracts. Based on the present data, it was decided to use the 5000 DPP/ml dose in further clinical development under the German Therapy Allergen Ordinance.^26^, ^27^

## AUTHOR CONTRIBUTIONS

**Oliver Pfaar**: Writing—original draft (equal); writing—review and editing (equal). **Angelika Sager**: Conceptualization (lead); formal analysis (lead); methodology (lead); project administration (lead); resources (lead); writing—original draft (equal); writing—review and editing (equal). **Ralph Mosges**: Writing—original draft (equal); writing—review and editing (equal). **Margitta Worm**: Conceptualization (supporting); investigation (supporting); Methodology (supporting); writing—original draft (equal); writing—review and editing (equal).

## CONFLICT OF INTEREST STATEMENT

Oliver Pfaar reports grants and/or personal fees from ALK‐Abelló, Allergopharma, Stallergenes Greer, HAL Allergy Holding B.V./HAL Allergie GmbH, Bencard Allergie GmbH/Allergy Therapeutics, Lofarma, ASIT Biotech Tools S.A., Laboratorios LETI/LETI Pharma, GlaxoSmithKline, ROXALL Medizin, Novartis, Sanofi‐Aventis and Sanofi‐Genzyme, Med Update Europe GmbH, streamedup! GmbH, Pohl‐Boskamp, Inmunotek S.L., John Wiley and Sons, AS, Paul‐Martini‐Stiftung (PMS), Regeneron Pharmaceuticals Inc., RG Aerztefortbildung, Institut für Disease Management, Springer GmbH, AstraZeneca, IQVIA Commercial, Ingress Health, Wort&Bild Verlag, Verlag ME, Altamira Medica AG, Meinhardt Congress GmbH, Deutsche Forschungsgemeinschaft, Thieme, Deutsche AllergieLiga e.V., AeDA, Procter and Gamble, Alfried‐Krupp Krankenhaus, Red Maple Trials Inc., Technical University Dresden, ECM Expo& Conference Management, all outside the submitted work; and he is member of EAACI Excom, member of ext. board of directors DGAKI; coordinator, main‐ or co‐author of different position papers and guidelines in rhinology, allergology and allergen‐immunotherapy. Angelika Sager is an employee of LETI Pharma GmbH. Ralph Mösges reports grants and personal fees from LETI Pharma within the scope of the present work. He also reports recent grants, personal fees, and non‐financial support (all outside the scope of the present work) from ALK, Allergopharma, Allergy Therapeutics, Angelini Pharma, ASIT biotech, Atmos, Bayer, Bencard, Bionorica, bitop, Cassella‐med GmbH and Co. KG, FAES, Ferrero, Friulchem, GSK, HAL BV, Hexal, Hulka, Inmunotek, JGL, Johnson&Johnson, Klosterfrau, Laboratoire de la Mer, Lek, Lofarma, Meda, Menarini, Menarini, Merck Sharp and Dohme, Mundipharma, Novartis, Nuvo, Optima, Otonomy, Pohl‐Boskamp, PRO‐AdWise, Roxall, Sanofi, Servier, Sidroga, Stada, Stallergenes, UCB, and Ursapharm. Margitta Worm declares the receipt of honoraria and/or consultation fees by the following companies: Novartis Pharma GmbH, Sanofi‐Aventis Deutschland GmbH, DBV Technologies S.A, Aimmune Therapeutics UK Limited, Regeneron Pharmaceuticals, Inc, Leo Pharma GmbH, Boehringer Ingelheim Pharma GmbH &Co.KG, ALK‐Abelló Arzneimittel GmbH, Lilly Deutschland GmbH, Kymab Limited, Amgen GmbH, Abbvie Deutschland GmbH and Co. KG, Pfizer Pharma GmbH, Mylan Germany GmbH (A Viatris Company), AstraZeneca GmbH, Lilly Deutschland GmbH, and GlaxoSmithKline GmbH and Co. KG.

## Supporting information

Supporting Information S1

## Data Availability

GCP conform The data that support the findings of this study are available from the corresponding author upon reasonable request.
