# Supplementary material for: A high‐dose, depigmented polymerized birch pollen extract for subcutaneous allergen immunotherapy has a favourable efficacy/safety ratio
Source: Clin Transl Allergy. 2023 Nov 15;13(11):e12315. doi: 10.1002/clt2.12315 (PMC10646866; doi:10.1002/clt2.12315)
Supplement: Supplementary file 1 — Supporting Information S1 [file CLT2-13-e12315-s001.docx]

Supplementary Material

Supplementary Table 1. Skin prick test positivity at V1 in each group (safety set, n=343).

| Allergen extract tested | Overall  (n=343) | 100 DPP/ml  (n=89) | 1000 DPP/ml  (n=81) | 5000 DPP/ml  (n=86) | 10000 DPP/ml  (n=87) |
| --- | --- | --- | --- | --- | --- |
| Birch pollen | 342 (99.7) | 89 (100.0) | 81 (100.0) | 85 (98.8) | 87 (100.0) |
| Phleum pollen | 91 (26.5) | 23 (25.8) | 19 (23.5) | 23 (26.7) | 26 (29.9) |
| Weed pollen | 86 (25.1) | 18 (20.2) | 23 (28.4) | 19 (22.1) | 26 (29.9) |
| Alternaria spores | 23 (6.7) | 6 (6.7) | 2 (2.5) | 9 (10.5) | 6 (6.9) |
| House dust mites | 74 (21.6) | 17 (19.1) | 18 (22.2) | 18 (20.9) | 21 (24.1) |
| Cat epithelium | 66 (19.2) | 21 (23.6) | 16 (19.8) | 17 (19.8) | 12 (13.8) |
| Dog epithelium | 85 (24.8) | 25 (28.1) | 19 (23.5) | 19 (22.1) | 22 (25.3) |

The data are quoted as n (%) positive tests (wheal diameter ≥3 mm or at least the size of the wheal for a histamine reference test). DPP: depigmented, polymerized birch pollen extract.

Supplementary Table 2: Baseline allergen-specific IgE in the groups (safety set, n=343).

|  | **Overall**  **(n=343)** | **100 DPP/ml**  **(n=89)** | **1000 DPP/ml**  **(n=81)** | **5000 DPP/ml**  **(n=86)** | **10000 DPP/ml**  **(n=87)** |
| --- | --- | --- | --- | --- | --- |
| IgE birch | 65.5 (34.71) | 65.5 (33.56) | 65.1 (35.31) | 64.2 (36.52) | 67.3 (33.98) |
| IgE *P. pratense* | 3.0 (7.26) | 2.9 (7.71) | 2.5 (5.61) | 2.7 (6.01) | 3.8 (9.12) |
| IgE *D. pteronyssinus* | 1.1 (4.66) | 1.0 (2.32) | 1.4 (7.05) | 1.5 (5.68) | 0.8 (1.59) |
| IgE *D. farinae* | 1.0 (3.79) | 0.9 (1.98) | 1.2 (5.80) | 1.3 (4.46) | 0.8 (1.37) |
| IgE cat epithelium | 3.6 (12.64) | 2.6 (7.37) | 4.9 (16.48) | 4.2 (12.95) | 3.0 (12.54) |
| IgE dog epithelium | 0.9 (5.97) | 0.5 (0.96) | 2.4 (12.17) | 0.4 (0.08) | 0.4 (0.08) |
| IgE moulds | 0.4 (0.44) | 0.5 (0.84) | 0.4 (0.09) | 0.4 (0.02) | 0.4 (0.17) |
| IgE grasses | 0.9 (3.60) | 1.0 (4.61) | 0.5 (0.39) | 1.1 (3.92) | 1.2 (3.78) |

The data are quoted as the mean ± standard deviation kU/L. DPP: depigmented, polymerized birch pollen extract.

Supplementary Table 3: Immunological endpoints: birch-specific serum IgG_1_ and IgG_4_ titres at V1 and V8, with V8-V1 differences.

|  | **100 DPP/ml** | **1000 DPP/ml** | **5000 DPP/ml** | **10000 DPP/ml** |
| --- | --- | --- | --- | --- |
| ITT population (n=343) | | | | |
| *Birch IgG_1_ (kU/L)* | | | | |
| V1 | 54.8 ± 93.92 | 59.2 ± 94.66 | 63.9 ± 169.78 | 62.4 ± 141.80 |
| V8 | 91.6 ± 175.03 | 112.5 ± 178.28 | 194.4 ± 288.20 | 199.6 ± 301.94 |
| V8-V1 | 44.0 ± 103.18 | 53.3 ± 141.07 | 162.5 ± 282.63 | 134.2 ± 191.42 |
| *Birch IgG_4_ (ng/mL)* | | | | |
| V1 | 4,432.1 ± 4,113.31 | 7,024.0 ± 6,626.48 | 7,000.0 ± 9,248.91 | 5,024.1 ± 6,160.11 |
| V8 | 6,206.9 ± 10,555.73 | 18,088.0 ± 25,640.74 | 24,552.0 ± 33,134.88 | 23,128.6 ± 29,265.96 |
| V8-V1 | 1,774.8 ± 7,505.45 | 11,064.0 ± 22,027.48 | 17,372.0 ± 26,124.39 | 18,250.0 ± 26,073.82 |
| PP population (n=301) | | | | |
| *Birch IgG_1_ (kU/L)* | | | | |
| V1 | 52.6 ± 92.08 | 48.4 ± 89.83 | 69.9 ± 188.41 | 65.4 ± 146.55 |
| V8 | 81.1 ± 169.16 | 88.1 ± 157.49 | 221.4 ± 308.54 | 207.5 ± 305.46 |
| V8-V1 | 37.0 ± 102.82 | 39.7 ± 137.42 | 191.0 ± 300.81 | 139.4 ± 193.48 |
| *Birch IgG_4_ (ng/mL)* | | | | |
| V1 | 4,525.2 ± 4,180.90 | 6,913.6 ± 6,850.67 | 8,066.7 ± 10,027.38 | 4,977.8 ± 6,318.07 |
| V8 | 6,624.0 ± 11,302.33 | 18,422.7 ± 27,311.50 | 28,666.7 ± 34,720.42 | 23,955.6 ± 29,488.18 |
| V8-V1 | 2,098.8 ± 8,040.57 | 11,509.1 ± 23,205.25 | 20,600.0 ± 27,388.30 | 18,977.8 ± 26,279.10 |

The data are quoted as the mean ± standard deviation. DPP: depigmented, polymerized birch pollen extract; V: visit. ITT: intention-to-treat, PP: per-protocol.
